# The complexity of scaling up an mHealth intervention: the case of SMS for Life in Tanzania from a health systems integration perspective

**DOI:** 10.1186/s12913-021-06285-8

**Published:** 2021-04-14

**Authors:** Carmen Sant Fruchtman, Selemani Mbuyita, Mary Mwanyika-Sando, Marcel Braun, Don de Savigny, Daniel Cobos Muñoz

**Affiliations:** 1 Swiss Tropical and Public Health Institute, Basel, Switzerland; 2 University of Basel, Basel, Switzerland; 3 Africa Academy for Public Health, Dar Es Salaam, Tanzania; 4 Novartis Social Business, Basel, Switzerland

**Keywords:** Digital health, Scale-up, Systems thinking, Case study

## Abstract

**Background:**

SMS for Life was one of the earliest large-scale implementations of mHealth innovations worldwide. Its goal was to increase visibility to antimalarial stock-outs through the use of SMS technology. The objective of this case study was to show the multiple innovations that SMS for Life brought to the Tanzanian public health sector and to discuss the challenges of scaling up that led to its discontinuation from a health systems perspective.

**Methods:**

A qualitative case-study approach was used. This included a literature review, a document review of 61 project documents, a timeline of key events and the collection and analysis of 28 interviews with key stakeholders involved in or affected by the SMS for Life programme. Data collection was informed by the health system building blocks. We then carried out a thematic analysis using the WHO mHealth Assessment and Planning for Scale (MAPS) Toolkit as a framework. This served to identify the key reasons for the discontinuation of the programme.

**Results:**

SMS for Life was reliable at scale and raised awareness of stock-outs with real-time monitoring. However, it was discontinued in 2015 after 4 years of a national rollout. The main reasons identified for the discontinuation were the programme’s failure to adapt to the continuous changes in Tanzania’s health system, the focus on stock-outs rather than ensuring appropriate stock management, and that it was perceived as costly by policy-makers.

Despite its discontinuation, SMS for Life, together with co-existing technologies, triggered the development of the capacity to accommodate and integrate future technologies in the health system.

**Conclusion:**

This study shows the importance of engaging appropriate stakeholders from the outset, understanding and designing system-responsive interventions appropriately when scaling up and ensuring value to a broad range of health system actors. These shortcomings are common among digital health solutions and need to be better addressed in future implementations.

**Supplementary Information:**

The online version contains supplementary material available at 10.1186/s12913-021-06285-8.

## Background

The accelerating role of electronic information and communication technologies (ICT) in low-and middle-income countries (LMICs) is seen as one of the major opportunities for the health sector [1]. Increased operational efficiencies, as well as low-cost deliveries, have led to many investments in digital solutions [2, 3]. There has been rapid evolution from mobile health (mHealth), which focussed mainly on the use of mobile phones, to electronic health (eHealth), or digital health, which also include further technologies, such as apps or web-based solutions [4, 5].

Despite the increased investment in digital health, few programmes have managed to be successfully upscaled in LMICs. District Health Information Systems (DHIS2) is one of these exceptions [2, 6]. The main drivers of success for DHIS2 have been described by its open-source software technology, its capacity to adapt to changes in technology and health systems, as well as the DHIS2 community which is built up around projects and activities from universities, NGOs and health authorities in LMICs [6]. It remains the case that digital health interventions are usually undertaken in stand-alone, vertical projects and target relatively small populations [2, 7] rather than integrated as part of the health system. As a result, the focus of digital health initiatives has shifted towards scalability, integration, and sustainability of programmes [7, 8]. This move has been driven by LMIC governments, partner agencies and donors aiming to move beyond the pilot stage and implement solutions that can be scaled up and run in the long-term [9].

The focus of this study is SMS for Life 1.0, a solution developed as part of a public-private partnership to address stock-outs (defined as a complete lack of a specific drug) in sub-Saharan Africa. SMS for Life was designed to make stock-outs in remote facilities more visible. The programme assumed that by showing national and district managers where stock-outs occurred, these actors would arrange the redistribution of drugs between facilities or organise an emergency delivery from the medical stores department (MSD) [10].

One health worker in each facility was trained to send weekly SMS with the counts of stock for four artemisinin-combination therapy (ACT) and quinine, which at the time were the first and second line antimalarials in the country [10]. The private phone numbers of the trained health workers were registered and activated in the SMS for Life system [11].

Reminders were sent automatically to the health worker’s phone. If the health worker responded within 27 h, they received credit on their phone (TZs 1000, equivalent to USD 0.70) for personal use (incentive) [12]. These SMS were free of charge thanks to an agreement with the mobile service provider. Weekly dashboards were automatically generated and sent to the District Medical Officer, to the National Malaria Control Program, other Ministry of Health, Community Development, Gender, Elderly and Children (MOHCDGEC) officials and the donors.

SMS for Life was piloted and scaled-up nationwide in Tanzania in 2011. The programme monitored weekly stock levels of the official first- and second-line antimalarials used countrywide in over 5000 government and non-governmental health facilities; it was one of the earliest large-scale mHealth innovations in LMICs. The programme ran for 4 years and was discontinued in 2015.

SMS for Life has been described in the literature before [10, 12]. Barrington et al. [10] presented the successful pilot results and Mikkelsen-Lopez et al. [12] showed that after 2 years of scale-up, health facilities continued to experience severe antimalarial stock-outs. Both papers studied the programme using a quantitative approach and did not explore the reasons behind the results. With this qualitative case study, we aimed to bridge this gap and understand why it was discontinued.

The framework we used to guide data collection is de Savigny et al’s building blocks which put people at the centre of the health system and show the interaction among the six building blocks: Medicines and Technology, Governance, Information, Financing, Human Resources, and Service Delivery [13]. By applying a systems lens to the SMS for Life case study we intended to examine the opportunities and challenges of scaling up and integrating a new electronic health technology into a complex health system.

The results of the study are presented using an adaptation of the building blocks framework, the WHO mHealth Assessment and Planning for Scale (MAPS) toolkit [14]. The MAPS toolkit was developed by WHO to help project managers that were implementing mHealth solutions, to plan and implement for scale. It compiles actionable information to self-assess projects when planning for scale or when scale-up is already achieved to assess the maturity of the programme.

The lessons learned in this study should be instructive for future digital solutions aiming to go to scale and integrate into similar settings.

## Methods

This was a qualitative case study of a nationwide mHealth implementation. Case study methodology allows us to look in detail at all aspects of the programme and extract the lessons learned from it [15]. To conduct this case study, we used the WHO health system building blocks framework [13] to guide data collection from a health-systems perspective and the WHO mHealth Assessment and Planning for Scale (MAPS) toolkit as a framework to analyse and interpret this data [14].

### Study setting

Tanzania’s public health supply chain system serves the MOHCDGEC-operated facilities, including 20 regional vaccine stores, 137 district stores, and 6821 service delivery points (hospitals, health centres, and dispensaries). The value of medicines and other commodities moving through the public health supply chain each year is roughly 300 billion Tanzanian shillings (TSh) (about 130 million USD at 2021 exchange rates) [16].

The MOHCDGEC has overall responsibility for ensuring the system remains functional. System logistics are run by the MSD, a semi-autonomous organisation in MOHCDGEC [16, 17]. At the district level, the forecast and ordering of commodities are done by district pharmacists, whose supervision lies under the President’s Office Regional Administration and Local Government (PORALG), as part of Tanzania’s decentralized system [18].

### Data collection

This case study was built on the triangulation of three data collection methods: document review, participatory observation and key informant interviews. A total of 61 documents, 3 participatory observations, and 24 interviews with 28 interviewees were included in the thematic content analysis.

We conducted a preliminary quantitative analysis to explore district performance in SMS for Life. We used the available data from a previous study, which contained SMS for Life reporting data from between June 2011 and December 2012 [12].

We classified districts by percentage of weeks in which stock was reported by the facilities and percentage of complete stock-out among those facilities that reported. In order to choose which districts would be included in the study, other descriptive factors were included in the tabulation: urban vs rural, pilot district vs non-pilot district and malaria prevalence in each district. Based on this preliminary analysis, NIMR approval and convenience to reach the study area, we chose Rufiji District in Coast Region and Dodoma Urban Municipality in Dodoma Region to conduct our district-level interviews.

### Document review

We reviewed published literature, unpublished reports, evaluations, memoranda of agreement, and other available project documents to gather information on inputs, outputs, and outcomes of the programme. A data extraction tool was developed to gather key information from these.

### Key stakeholder interviews

Two main groups of participating stakeholders were identified, those involved in high-level governance and design of the programme and those at the frontline level (district officials and health workers). Based on our preliminary performance analysis, two districts were purposively selected to conduct interviews, a high and a low-performing district. Purposive and snowball sampling were used to select the interviewees. Interview guides were developed to conduct semi-structured interviews with each group (available as an Annex). These guides were structured following the WHO health system building blocks [13]. Probes were used to elicit additional information based on the course of conversations and interviewer’s observations.

CS, a female master’s student of public health from Spain conducted all interviews. DS, a Canadian male professor in health systems, accompanied CS in the interviews conducted in Switzerland and SM, a male senior social scientist holding a Master of Public Health from Tanzania co-led the interviews in Tanzania. SM led the introductions to the interviewees. Information about the study aim was shared with all participants and written or oral informed consent was collected.

Informed consent was provided by 28 interviewees (21 male, 7 female). Three additional interviews were aimed at the district and national level, but the interviewees argued having no time for the interview or the phone number provided didn’t work. Table 1 describes the organization and level of these, defined by the geographical reach of their work.

**Table 1:** Work organization and level of interviewees

Stakeholder organisation	Level	Interviewees
Civil society	National	2
District Health Management Team	District	3
Donor SMS for Life	Global	3
External advisor Ministry of Health	Global/National	2
External researcher	National	2
Health personnel reporting with SMS	Health facility	3
Medical Stores Department (MSD)	National	1
Ministry of Health	District	3
National Malaria Control Programme	National	3
Other supply chain donors	Global/National	2
SMS for Life project management	Global/National	4

Most interviews [19] were conducted face-to-face in the place of work of the interviewees. A confidential and quiet place was always found to discuss the study questions. Two participants were interviewed over the phone by CS and SM from Tanzania and five interviews were conducted over Skype by CS from Switzerland. The average duration of these interviews was 1 h. Three interviews were conducted with two interviewees simultaneously. No interview was repeated. Sampling was stopped once data saturation was reached, as described by Ando et al. [20].

### Participatory observations

The participatory observations were conducted by CS and SM. The objective of this was to understand how drugs are stored in the dispensary, ordered at the health facility and dispensed by MSD. The data collected during these observations were summarised and allowed the researchers to further understand the drug delivery processes.

### Data analysis

Interviews were audio-recorded, transcribed and translated, when necessary, into English. Researchers’ interview notes were typed and organised into summaries of each interview. This collation encouraged immersion in the data to aid analysis and reflection from an early stage during data collection. CS and SM both participated in this process.

Transcripts and interview summaries were shared, when possible, with the study participants. Nevertheless, no feedback was received from these.

All interviews were coded and analysed by CS, who was supervised by DC and DS. CS conducted a deductive thematic analysis of the transcripts and collated documents (Table 2) in Nvivo12 software. The codebook had been previously developed using the WHO MAPS toolkit. Findings were validated by the wider research team.

**Table 2:** Domains explored during thematic content analysis using MAPS toolkit

MAPS Domain	Theme
Groundwork	Were the policy and technical environments in the country assessed before scale-up?
Partnerships	Were specific product champions established among core partners?
Financial health	Were the potential economic costs of scaling up the technology forecasted?
Technology & architecture	Were relevant steps taken to ensure interoperability with relevant information systems?
Operations	Were training programmes developed to train end-users and secondary users?
Monitoring & evaluation	Were appropriate resources allocated to monitoring and evaluation during scale-up?

### Ethical issues

The study received approval from ethics committees at the Swiss Tropical and Public Health Institute, the Tanzania IRB (NIMR/HQ/R.8a/Vol.IX/2767) and COSTECH. All participants in the study gave informed verbal consent after being given a written study information sheet and a verbal explanation of the consenting process. Verbal informed consent was seeked to maintain the informal raport during the interview. This was approved by the ethics committees.

## Results

We used the domains from the WHO MAPS toolkit to structure the main findings of our qualitative study (summarised in Table 3, [14]). The high costs of the programme (50′000 USD/month), combined with its focus on a single disease, its failure to adapt to the arrival of competitor systems to Tanzania, and the lack of continuity between ministerial leaders, were shown to be the main reasons for its discontinuation.

**Table 3:** Summary of main themes identified in the thematic analysis across the MAPS domains

MAPS Domain	Main themes identified	
	Positive	Negative
Groundwork	➢ Very successful pilot ➢ The goal of the programme aligned with the country’s priorities	➢ SMS for Life was short in objective-setting (focus on stock-out visibility and not on an appropriate response to stock-outs)
Partnerships	➢ SMS for Life aimed to engage with all key stakeholders	➢ Not all key stakeholders were involved in the design and deployment (e.g district pharmacists) ➢ Narrow partnership scope focussed on limited commodities for a single disease control programme ➢ No inclusion of Zonal or National MSD in the system ➢ Lack of ownership at MSD and district level led to a lack of action when stock-outs were reported ➢ Changes in government, ministerial leadership and political agendas led to the loss of SMS for Life’s momentum ➢ This loss of momentum was strongly influenced by the arrival of competitor mHealth solutions
Financial health	➢ SMS for Life project managers described the system as a very low cost solution as it didn’t need any maintenance or technical support	➢ The costs of the programme were stated as one of the main reasons for discontinuation although no real costing was done ➢ It was difficult for the government to allocate a substantial share of its budget to malaria commodities and not to others ➢ Main drivers of the programme’s costs were: SMS and health worker incentives (pay-for-performance)
Technology & architecture	➢ Real-time data in rural health facilities in a time when this was uncommon ➢ Described as a user-friendly system ➢ Technology included quality checks ➢ Interoperability within mobile network operators was an innovation and at the same time challenge for the programme ➢ Technology was an innovation for Tanzania and mHealth globally	➢ Lack of adaptability of the technology to new circumstances in the country ➢ Data server and hosting in the United Kingdom seen as a political challenge ➢ Lack of interoperability with the national health management information system (HMIS) ➢ No change or improvement in the user interface between 2009 and 2015
Operations	➢ SMS for Life trained health facility personnel to store commodities more efficiently, these changes are still visible today	➢ The scale-up planning only included the organization of training ➢ Lack of response from the health system in case of stock-outs (see Table 4) ➢ High turnover of personnel at the health facilities caused a knowledge loss of the programme, as only one person was trained per facility. This led to high attrition rates from the system ➢ No continuing training plans
Monitoring & evaluation	➢ Antimalarial stock situation improved during the years, mainly due to other changes in Tanzanian supply chain system (e.g. MSD Direct Delivery system).	➢ No monitoring and evaluation framework ➢ One external evaluation conducted in 2013 reported several challenges that were never addressed

### Groundwork

Novartis and IBM organized a joint event to find innovative solutions to address stock-out challenges in sub-Saharan Africa [10]. Twenty students from all over the world joined forces during 12 weeks in Basel, Switzerland and mapped drug supply chain systems in sub-Saharan Africa and investigated possible improvements of this. Novartis chose one of the suggested solutions for further development, an SMS-based stock management solution [19]. This technology was then further developed in a partnership by Novartis, Google, IBM and Roll Back Malaria (RBM) and would eventually become SMS for Life.

Tanzania was selected as the country where this system would be tested first. The reasons mentioned to choose this country were its struggle to ensure availability of medicines, among them antimalarials and strong bonds of some SMS for Life partners with the country [21].

Within this case study, we explored how well the programme aligned with the country’s needs. Most interviewees expressed that SMS for Life was well aligned with the country’s priorities when it was implemented because the malaria burden was high and delivering first-line drugs was a challenge for the country. However, the interviewees stated that focussing on stock-outs and not on appropriate stock management fell short of solving the problem and caused some political challenges as no-one wanted to report bad news.

*“Sometimes people don't like to have bad news you know. If you see that you don't have stocks and maybe there is nothing that you can do centrally at MSD, you see. What do you do? You should just shout there are no medicines? Nobody wants to hear that.” National Malaria Control Programme*

### Partnerships

In terms of partnerships, SMS for Life aimed to engage the right partners before rolling out the programme. Its internally-approved, not publicly available implementation guidelines stated that: “before the SMS for Life project is rolled out in any country, it is vital that the key stakeholders involved in Malaria Prevention and Control, and the relevant people responsible for drug distribution and management are informed about, and involved in, the project.” However, the opinions of the interviewees differ. A substantial number of interviewees mentioned that not all key stakeholders were involved in the design and deployment of the programme. Some examples given that were not engaged were district pharmacists, the Medical Stores Department or officials at the regional level.

*“The programme should have been not only vertical but also horizontal and should have included the PO-RALG and MOH. Both should have been actively involved and not only passively aware of it” External researcher*

SMS for Life had strong champions during the pilot, but there were many changes in the government during the scale-up, which led to changes in priorities.

*“Changes in leadership, changes in the background in the people in charge, changes in the priorities, this all influences sustainability of programmes.”* District Medical Officer

In addition, shortly after the scale-up of SMS for Life, other supply chain information systems entered the Tanzanian landscape, including ILS Gateway. This programme had a similar objective and was also an SMS-based solution, but included 20 different products and had lower running costs. However, users reported this second system was more difficult to use.

The coexistence of both programmes with similar objectives but funded by different donors led to competition between them, even if thought to be complementary. ILSGateway and SMS for Life were making each other redundant. In a Global Fund Audit, the following was reported: “Although developed for different purposes and meant to be complementary, some of the developed systems have duplicative functions, e.g. the SMS4life and ILS gateway (costing USD 600,000 and 60,000 annually, respectively) collect similar information. There are plans to discontinue SMS4life under the new grants.” [22].

Several information systems were implemented since SMS for Life was scaled-up. MSD works with Epicore 9 to track the logistics of their procedures, while the health system has had different independent systems to track stock since the pull system was implemented: Request&Requisition (R&R) paper forms, end-use verification forms, tracer forms, ILS Gateway, DHIS2, and, most recently, eLMIS [23].

**Fig. 1:**
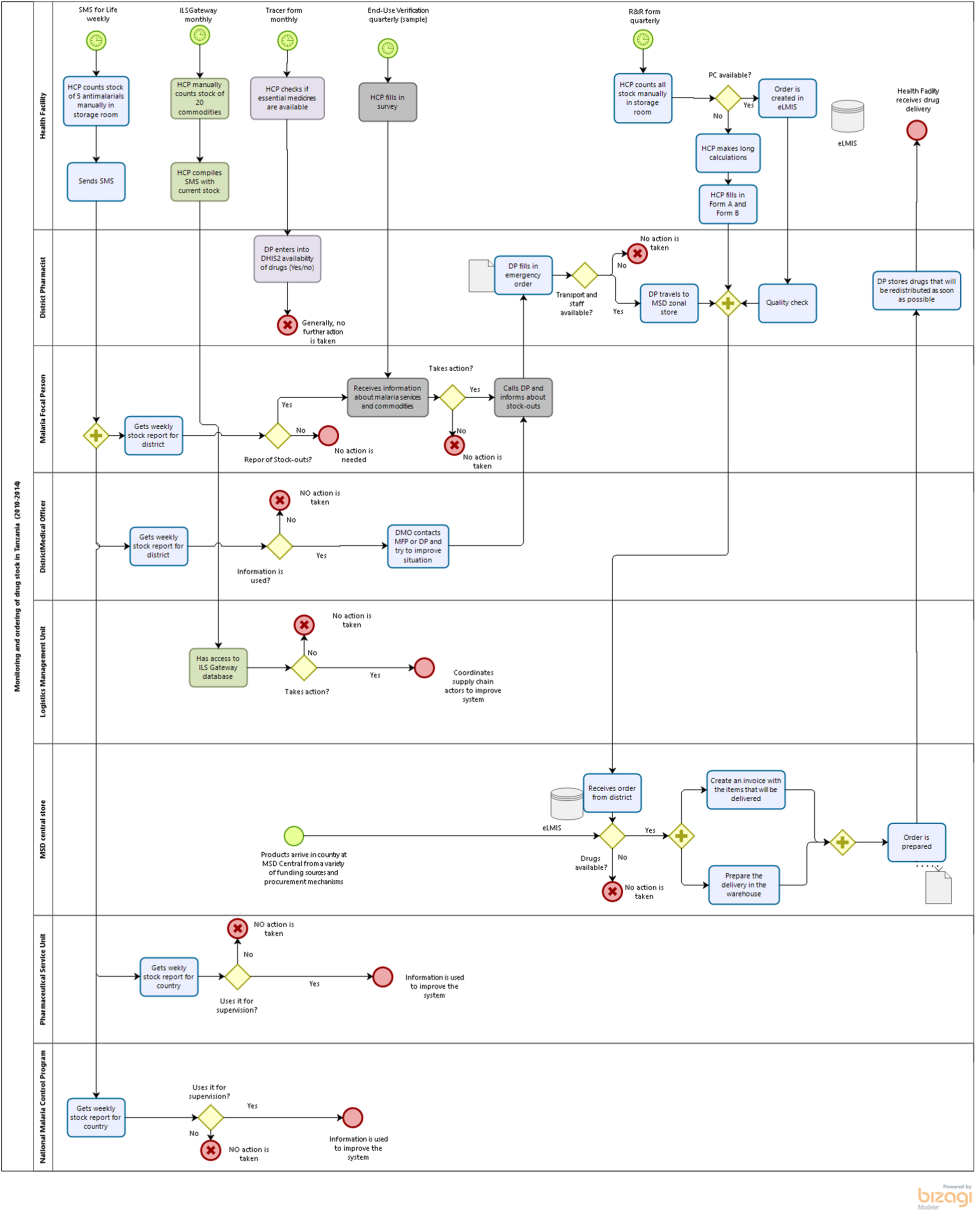
Process map representing parallel logistic systems in Tanzania (2011–2014)

These systems increased the complexity of taking action against stock-outs, as they were often reporting contradictory results (see process map in Fig. 1). To harmonize Tanzania’s digital systems, including the supply chain information systems, the country launched its national e-health strategy in 2013 [23].

### Financial health

With leadership changes, questions were also raised regarding the costs of the programme. Most interviewees agree that the main reason for discontinuation was that the programme was too expensive to cover just one disease. Interviewees described the difficulty to justify why a substantial share of the MoH’s budget should be dedicated to the supply chain of one programme and not others.

*“The system was very expensive. The government asked itself should I pay for a system that costs over 500`000 USD/year or buy drugs for that amount of money?” External researcher*

The programme was initially funded by external donors (Swiss Development Cooperation and Medicines for Malaria Venture). The MOHCGDEC had committed in their Memorandum of Understanding to take over the project management and the costs of SMS for Life once initial implementation had been set up. However, as the agreed date for this was approaching (January 2012), the government requested more time to prepare for the take-over. The following year the government asked for another extension of funding to MMV. The organization agreed to do so until the end of March 2013. From April 2014, the costs for running the programme were covered within a Global Fund grant to the Tanzanian government.

*“This was an added challenge for the programme and its continuity because all funders have different horizons. Every year it was necessary to go back and find a new funder. Somehow resell the program. All the funds were coming from the EU.” Mobile operator*

The programme costs reported per month differ in the reports and range between 47,000 and 55,300 USD/month.

### Technology & Architecture

Despite some challenges being identified in the political arena, the programme was well-received among health facility personnel and it demonstrated that health facilities could use SMS technologies easily and provide continuous stock reporting [24]. Facilities only had to enter the first letter of the colour of the ACT and the number of stocks for each.

*“I was surprised by how good the health facilities understood the system. They really knew how to send those long SMS and were really patient if something was not working.” Programme evaluator*

### Operations

The simplicity of sending a weekly SMS was a contrast to the complexity and delays of responding to stock-outs using the quarterly paper system. Interviewees at the highest level of the national institutions explained that the response to stock-outs was a big challenge for SMS for Life. Health facilities were reporting stock-outs, but the information reported did often not trigger a response to solve these. The reasons for the lack of response when a stock-out was reported through SMS for Life are shown in Table 4.

*“It was all obviously visibility. Everybody could see that. Places were running low on malaria medicine, but the action was not, was not always there. Maybe we could not sometimes solve the problem. Sometimes these drugs were shipped from overseas and they were not in the stores. Sometimes the drugs were at the stores, but the district could not go and pick them up. Sometimes. Even with the district itself, you could see a facility that has stocks but moving a drug from where you come from to here required... because sharing was not always easy. If I have got an extra stock at time x and I'm not sure when I'm going to get the replenishment, you know I cannot share with you, because I don't know if I’ll get the replenishment over the next months. That was also the challenge, but otherwise, the success was that it was possible to transmit this information.” National Malaria Control Program*

**Table 4:** Reasons stated for the lack of action to solve stock-outs at district level

1) Commodities claimed not to be available at MSD, often due to delays in procurement	
2) Lack of resources for transport between health facilities or to pick up drugs at MSD	
3) Unwillingness by the health facilities to reallocate their surplus stock due to lack of confidence in a replenishment delivery	
4) Lack of ownership of district leadership of the programme, which led to a lack of use of the collected data	

### Monitoring & evaluation

Despite the challenges raised with SMS for Life response, the evidence found, including the WHO MoH Service and Availability Readiness Assessment (SARA), showed that antimalarial stock-outs were occurring less frequently in most parts of the country during the time of SMS for Life, especially from 2014 [22, 25, 26]. The main reason stated by most interviewees for this improvement in stocks was not SMS for Life, but modifications in the procurement system that were occurring in parallel. Vertical programmes (e.g. national malaria control programme) and health facilities were allowed to receive funds directly to purchase drugs, instead of having to go through the MSD. The purchase of drugs was decentralised and direct delivery was introduced. As part of this substantial initiative to improve the stock situation, the electronic Logistics Management Information System (eLMIS) was launched and the Logistics Management Unit (LMU), within the MoH, was created to manage the system [22].

## Discussion

The overarching purpose of this study was to extract the lessons learned of SMS for Life, which should serve as learnings for the design and implementation of future programmes. We aimed to contribute to a growing body of literature examining digital health interventions, including the challenges involved in integrating and sustaining such interventions in a complex health system.

We chose to study SMS for Life 1.0 because it was one pilot that had managed to overcome the initial challenges and reached full national scale in 2011, a time when digital health was still emerging in LMICs and the majority of pilots failed to reach this scale [1, 27]. However, despite initial success, the programme was discontinued without a final evaluation in 2015.

During the rollout, the technology of SMS for Life proved to be technically reliable and well appreciated. The programme collected real-time data in rural health facilities at a time when this was uncommon [10] and ultimately achieved its stated primary objective to provide visibility of antimalarial stock-outs.

Our analysis suggests that this narrow objective fell short of what was required in Tanzania, reducing stock-outs. The programme would have been improved by aiming to reduce stock-outs, rather than just giving visibility to these. A change in aim would have encouraged not only the reporting but also the action necessary to solve stock-outs by the health system. The literature describes this output-driven set-up as a common shortfall of digital health projects being implemented around 2010 [28]. In 2011, and as a response to this general challenge, the WHO Bellagio Statement on eHealth Evidence was written, calling for better evaluation and research of digital health programmes [29].

SMS for Life was built within the national malaria control programme and failed to consolidate and communicate with other programmes or departments (e.g. MSD, HMIS Department) [30–32]. In the early days of digital health, this was a common shortcoming where many digital health solutions were developed in isolation and focussed on a single challenge [30, 33, 34]. For future digital health programmes, identifying a process that could benefit more than one healthcare problem, would ensure greater stakeholder buy-in and set up the programme for prolonged success.

Our data shows that during the scale-up of the programme, many internal changes occurred in Tanzania that impacted the sustainability of SMS for Life. These included changes in government, treatment policies, and supply chain systems. One of the major changes was the introduction of new logistics management information systems. At the health facility level, the coexistence of different systems with similar objectives confused the health personnel and at the national-level it caused competition between programmes.

With time, champions of SMS for Life transitioned to other government positions and thus support for the programme’s implementation waned. Meanwhile, the ILS Gateway gained relevance in the new government. ILS Gateway was perceived as a more cost-effective solution, although an economic evaluation was never conducted to compare both systems. Despite this, most interviewees considered SMS for Life a more reliable technology. Iribarren et al. [35] report this lack of economic evaluation and comparison within programmes in their systematic review and advocate for the use of economic methods to undertake informed decisions. The more recent digital health guidelines published by WHO also highlight the need to conduct detailed costing exercises [36].

In Tanzania, the MoH had initially committed to taking over the costs of SMS for Life but this never transpired. When it came to taking these costs into its own budget, the programme was discontinued. This has been reported before, as there is often a large gap between the initial commitment and the actual realisation of assuming the costs [28].

Additionally, SMS for Life lacked a monitoring and evaluation scheme, and no implementation research was conducted to assess the performance after scale-up. The WHO digital health guidelines also address this and call for more effectiveness studies to monitor performance and behavioural performance or user engagement for the benefit of future programmes [36].

Agile management techniques are now gaining momentum and we believe SMS for Life would have benefited tremendously from their application to adapt to the on-going changes in Tanzania.

### Implications for future digital health solutions at scale

**Fig. 2:**
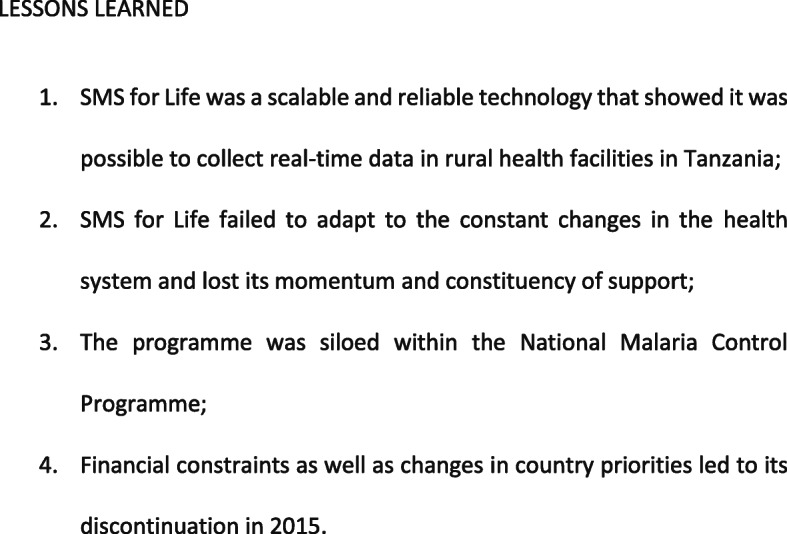
Lessons learned of SMS for Life scale-up in Tanzania

This study highlights the need to rethink interventions when going to scale and to highlight these in the literature for future implementation processes to learn from. The experience of SMS for Life is an important example of how not addressing system-wide factors in program design and implementation can lead to outdated and redundant programmes. We summarized the main lessons learned from the scale-up of SMS for Life in Fig. 2. We encourage the use of the MAPS toolkit for project managers as a good starting point to provide them with resources to assess the maturity of their mHealth programme and stage-based strategies for scale-up [14]. Agile project management schemes [37], cost evaluations and system approaches are additionally important tools to complement the toolkit in achieving sustainable programmes [38].

### Limitations

There is a level of subjectivity involved in qualitative case studies and this should be considered when interpreting the study findings. By applying different methodologies, we aimed to triangulate results and overcome some of the limitations associated with each methodology.

When conducting key informant interviews, the bias that has to be considered is of relevance. To start with, we should discuss the respondent’s bias. Most in-country interviews were conducted by a Tanzanian research scientist and the international investigator. The fact that the international investigator was of European origin could have influenced the responses of the interviewees, who could associate this with the programme management behind SMS for Life and affecting the honesty of responses.

In two cases, the interview had to be conducted with two respondents together. Researchers tried to encourage independent views, but it is not to exclude that the answers of one respondent affected the other.

## Conclusions

It is important to recognise the many successes and innovations that SMS for Life brought to Tanzania and to mHealth globally. The programme developed and introduced an SMS-based solution to address the serious antimalarial stock-out problem. However, it failed to address complex systems and individual, interpersonal, and institutional factors in programme planning, which ultimately led to its discontinuation.

To bridge this gap, we suggest the use of holistic methodologies and detailed costing exercises. These would help governments to carefully plan the programme to ensure its affordability right from its early stages. The programme would also have benefited from a health systems approach that would have guided programme managers to better navigate complex adaptive health systems [39, 40]. These learnings are echoed in the steps from the WHO view of systems thinking and the most recent WHO digital health guidelines [13, 36].

This research may be relevant to policy and decision-makers as well as programme planners, considering the expansion of digital health programmes to regional or national levels of service. We strongly suggest making use of the tools now available and include scale-up frameworks from the beginning of the intervention design.

Finally, despite the discontinuation of SMS for Life 1.0, key learnings can be taken forward to ensure the success of future programmes and ultimately improve healthcare for all.

## Supplementary Information

**Additional file 1.**

## Data Availability

The transcripts that were used for the development of this manuscript can be requested from the corresponding author.

## Abbreviations

ACT: Artemisinin-combination therapy
eHealth: electronic health
eLMIS: electronic Logistics Management Information System
HMIS: Health management information system
ICT: Information and communication technologies
LMICs: Low-and middle-income countries
MAPS: WHO mHealth Assessment and Planning for Scale
mHealth: mobile health
MOHCDGEC: Ministry of Health, Community Development, Gender, Elderly and Children
MSD: Medical stores department
PORALG: President’s Office Regional Administration and Local Government
SARA: Service and Availability Readiness Assessment
TSh: Tanzanian shillings
