# Supplementary material for: The complexity of scaling up an mHealth intervention: the case of SMS for Life in Tanzania from a health systems integration perspective
Source: BMC Health Serv Res. 2021 Apr 14;21:343. doi: 10.1186/s12913-021-06285-8 (PMC8048333; doi:10.1186/s12913-021-06285-8)
Supplement: Supplementary file 1 — Additional file 1. [file 12913_2021_6285_MOESM1_ESM.docx]

# ANNEXES

## Interview Guideline (Key Stakeholder)

Participant ID No |__|__|__|__| Gender Male / Female

Interviewer Initials |__|__|__| Date |__|__/__|__/__|__|

Country ______________

**Introduction**

I am ______________________________ from ______________________

- The goal of the case study is to perform a critical analysis with a health systems perspective of the lessons learned from SMS for Life 1.0 in Tanzania. This could lead to developing guidelines for the implementation of SMS for Life 2.0.
- The aim of this interview is to collect all the information you can provide us with the implementation of the programme SMS for Life. This will allow the evaluators to draw the lessons learned from it. The expected duration of this interview is from 45- 60 Min.
- We will interview you and all relevant stakeholders from international to the district level.
- We really appreciate that you take the time for this interview. Your inputs, thoughts and opinions will be very valuable to understand what were the strengths and weaknesses of SMS for Life.
- The information you provide us will be coded and saved confidentially. Only the evaluators will be able to identify you with the information provided. If any of the information you provide is to be published, we will make sure that this is strictly anonymous.
- Any questions?
- Consent

Interviewee’s signature ____________________________

**Warm up [demographic & work history]**

Can I ask some details about you and your job?

1. Current Job Title ____________________________
2. Job title during SMS for Life __________________________
3. Highest Educational Grade attained _________
4. Years involved with SMS for Life:
5. What was your role in the programme SMS for Life?
6. According to you, what were the goals of this programme?
7. How did these goals relate to the priorities in your district/region at the time?
8. How did you perceive the stock situation?
   1. Specifically antimalarial stockouts? Why?
9. What kind of impact do you think SMS for Life had?
   1. Prompt: Why did this change with the programme?

**Information**

1. When did you first learn about SMS for Life? By whom?
2. What kind of information did you receive once the programme was started?
   1. Prompts: And when it was running? And once it finished?
3. With which other stakeholders were you in contact?
   1. How often? What kind of exchange did you have?
4. What was your perception of this information exchange?
   1. With which other stakeholders would you have liked to exchange? In which manner?
5. Who had access to the stock database?
   1. What kind of access did you have?
6. How often did you look at the stock out reports?
   1. Who else had access and/or was looking at these reports?

**Medicines & Technology**

1. How did you perceive the technical functioning of the SMS for Life system?
2. In your opinion, how was the system working after reporting of stock-outs?
3. How did the reporting change during the program?
   1. Why?
4. What were the challenges/ difficulties you experienced to implement this system?

**Human Resources**

1. What was the training the people involved in SMS for Life received?
   1. By whom? How did you perceive the quality of the training? Was it useful?
2. What did you find good/negative from the training?
3. What kind of relationship do you have with the people working at the health posts?
   1. And at the national/regional/district level? Do you communicate often?
4. How did you perceive the workload of the people working at the health posts?
5. In your opinion, was the system well received at health facility level? And district?

**Service Delivery**

1. What were the changes in antimalarial drug availability after the implementation of the system? In your opinion,, this is due to what?
2. Did you experience any other changes during the implementation of SMS for Life? In other diseases, products, HCP behaviour?

**Finance**

1. Who were the major donors for this project?
   1. Prompts: Did the funding change during the project? Was it as planned?
   2. How much was your organization funding?
2. This programme included a pay for performance scheme in which you received a payment after every SMS. What is your opinion about this system? How do you think it influenced the results of the programme?

**Governance**

1. In your opinion who was accountable for the project?
   1. Who should have been? Any difference in perception?
2. Do you think the different stakeholders had the same expectations of the programme?
3. In your opinion, were these expectations met?
4. With which other stakeholders did you collaborate during the programme?
   1. How? How often?
5. In which decision-making processes of the programme were you involved?
   1. Prompt: In which phase? who else was involved?
6. Did you have access to SMS for Life key documentation?
   1. Prompts: What kind? Who else did?
7. How did you perceive the communication within programme management?
   1. Which channels were used? Was everyone involved that should have?
8. Do you know why and when the programme ended?
   1. Who were the decision makers?
   2. What were the actions taken to roll out the programme?
   3. How was the sustainability of the programme considered?
9. In your opinion, what was working well in the programme? And what should have been done differently?

**Close up**

1. Do you have any further comments or thoughts you would like to share with us?

Once again thank you very much for your participation...

## Interview Guideline (Health Facility)

Participant ID No |__|__|__|__| Gender Male / Female

Interviewer Initials |__|__|__| Health facility number |__|__|

Date |__|__/__|__/__|__|

**Introduction**

I am ______________________________ from ______________________

- The goal of the case study is to perform a critical analysis of the lessons learned from SMS for Life 1.0 in Tanzania. This could lead to develop guidelines for the implementation of SMS for Life 2.0.
- The aim of this interview is to collect all the information you can provide us with the implementation of the program SMS for Life. This will allow the evaluators to draw the lessons learned from it. The expected duration of this interview is from 45- 60 Min.
- We will interview you and all relevant stakeholders from international to the district level.
- We really appreciate that you take the time for this interview. Your inputs, thoughts and opinions will be very valuable to understand what were the strengths and weaknesses of SMS for Life.
- The information you provide us will be coded and saved confidentially. Only the evaluators will be able to identify you with the information provided. If any of the information you provide is to be published, we will make sure that this is strictly anonymous.
- Any questions?
- Consent

**Warm up [demographic & work history]**

Can I ask some details about you and your job?

1. Current Job Title ____________________________
2. Job title during SMS for Life __________________________
3. Highest Educational Grade attained ___ __
4. Years worked at this facility |__|__|yrs|__|__|mths
5. What was your role in the program SMS for Life?
6. What were the main priorities at your health facility or district ?
   1. Did SMS for Life align with these? How important were stock outs for you? Were you aware of stock out problematic?
7. How many workers were at the health facility during that time?
8. Who was in charge of sending the SMS?
   1. How was this decision taken?
9. How is the healthcare seeking behaviour in malaria patients in your health facility?
   1. Do you think drug availability has anything to do with healthcare seeking behaviour?

**Information**

1. When did you first learn about SMS for Life?
   1. Prompt: By whom?
2. What were the other information systems besides SMS for Life for stock reporting at the time?
   1. Prompt: Which one? How was it for you to work with different reporting systems of the same product? What were the advantages/disadvantages of the different systems?
   2. Were these different systems integrated? Would that have been of help?
3. How did you learn about the stock out rates in your facility or district?
   1. Prompt: How often?

**Medicines & Technology**

1. How do you order drugs nowadays?
   1. How was it during SMS for Life time?
   2. Could you rely on the amount of ordered drugs arriving as ordered?
      1. If not, why?
2. What did you do if you run out of any essential drug?
3. How often did you report the stocks in your facility through SMS for Life system?
4. What was the response of the system for stock-outs?
5. Did the reporting change during the program? Why?
6. What were the challenges/ difficulties you experienced to implement this system?

**Human Resources**

1. What was the training you and your colleagues received on how to report to SMS for Life?
   1. Prompt: By whom? How did you perceive the quality of the training?
2. How many times were you trained?
3. What did you find good/negative from the training?
4. How did SMS for Life influence your workload?

**Service Delivery**

1. What was the change in antimalarial drug availability after the implementation of the system?
2. What were other changes you experiences during the implementation of SMS for Life? In other diseases, products, HCP behaviour?

**Finance**

1. This program included a pay for performance scheme in which you received a payment after sending every SMS. How did you perceive these payments?
   1. Were these on time?
2. Was your motivation affected by these payments? How? Would you or your staff have sent these SMS without receiving a payment?

**Governance**

1. Who do you think was responsible to run the program?
2. Do you think the responsible staff of the program had clear objectives?
3. In your opinion, what was working well in the program? What should have been done differently in the implementation?

**Close up**

1. Do you have any further comments or thoughts you would like to share with us?

Once again thank you very much for your participation...
